# Clinical Application of Novel Therapies for Coronary Angiogenesis: Overview, Challenges, and Prospects

**DOI:** 10.3390/ijms22073722

**Published:** 2021-04-02

**Authors:** Mohamed Sabra, Catherine Karbasiafshar, Ahmed Aboulgheit, Sidharth Raj, M. Ruhul Abid, Frank W. Sellke

**Affiliations:** 1 Cardiovascular Research Center, Rhode Island Hospital, Providence, RI 02903, USA; msabra1@lifespan.org (M.S.); ckarbasiafshar1@lifespan.org (C.K.); ahmad_aboul_gheit@brown.edu (A.A.); ; ruhul_abid@brown.edu (M.R.A.); 2 Division of Cardiothoracic Surgery, Alpert Medical School of Brown University, Providence, RI 02903, USA; sidharth_raj@brown.edu

**Keywords:** angiogenesis, gene therapy, stem cells, extracellular vesicles, clinical trials, bioengineering

## Abstract

Cardiovascular diseases continue to be the leading cause of death worldwide, with ischemic heart disease as the most significant contributor. Pharmacological and surgical interventions have improved clinical outcomes, but are unable to ameliorate advanced stages of end-heart failure. Successful preclinical studies of new therapeutic modalities aimed at revascularization have shown short lasting to no effects in the clinical practice. This lack of success may be attributed to current challenges in patient selection, endpoint measurements, comorbidities, and delivery systems. Although challenges remain, the field of therapeutic angiogenesis is evolving, as novel strategies and bioengineering approaches emerge to optimize delivery and efficacy. Here, we describe the structure, vascularization, and regulation of the vascular system with particular attention to the endothelium. We proceed to discuss preclinical and clinical findings and present challenges and future prospects in the field.

## 1. Introduction

Despite substantial efforts aimed at evidence-based optimization of standards for prevention and early management, ischemic heart disease (IHD) accounts for nearly 9 million of the 18 million cardiovascular disease (CVD) deaths worldwide in 2015 [1]. The advent of therapeutic interventions and diagnostic methods have undoubtedly improved outcomes and lowered overall mortality associated with IHD. Current management of this disease comprises revascularization by means of percutaneous coronary intervention or surgical bypass grafting, in addition to pharmacological interventions aimed at mitigating risk factors, such as hypertension and dyslipidemia, in concert with correcting myocardial oxygen supply/demand mismatch. However, a common challenge encountered by clinicians is the management of advanced and diffuse multivessel disease state resistant to conventional treatment modalities, therefore, requiring the study of novel therapeutic strategies.

The term angiogenesis was first used in 1935 by Arthur Hertig to describe the formation of new blood vessels in the placenta. It was not until 1971, however, when Folkman showed that solid tumors were able to extensively vascularize their core by inducing growth of new blood vessels from contiguous vasculature of normal tissue [2]. This observation initially prompted the idea that inhibition of tumor angiogenesis could be a potential anti-neoplastic therapeutic strategy. Soon thereafter, it became evident that induction of this mechanism of autonomous blood vessel growth in tissue subject to chronic ischemia, such as the myocardium or extremities, may provide collateral blood supply and preserve viability. This notion prompted interest in the molecular mechanisms of blood vessel growth to guide future therapeutic targeting.

By the early nineties, many key angiogenic factors were characterized including various isoforms of vascular endothelial growth factor (VEGF) and fibroblast growth factor (FGF). The data from studies evaluating intramyocardial administration of recombinant growth factors in animal models of myocardial ischemia were promising, providing impetus for many clinical trials evaluating the safety and efficacy of administration of these growth factors in patients with ischemic heart disease (IHD) [3,4]. However, with increased sample sizes and extended follow-up times post-therapy, it became evident that the positive effects of new vessel growth and functional improvement were short-lived and did not culminate in sustainable long-term benefit [5]. Additionally, the incidence of adverse effects such as tissue edema with local administration of these growth factors raised concern. Since the early trials, much effort has focused on identifying issues that hinder the efficacy of novel angiogenic therapies in the clinical setting. This review will shed light on recent advances in therapeutic angiogenesis in animal models and the challenges of clinical applications of these strategies. We further review the biology of angiogenesis, summarize preclinical and clinical findings, and describe a number of translational challenges and novel angiogenic strategies.

## 2. Structure of the Vasculature

**Figure 1:**
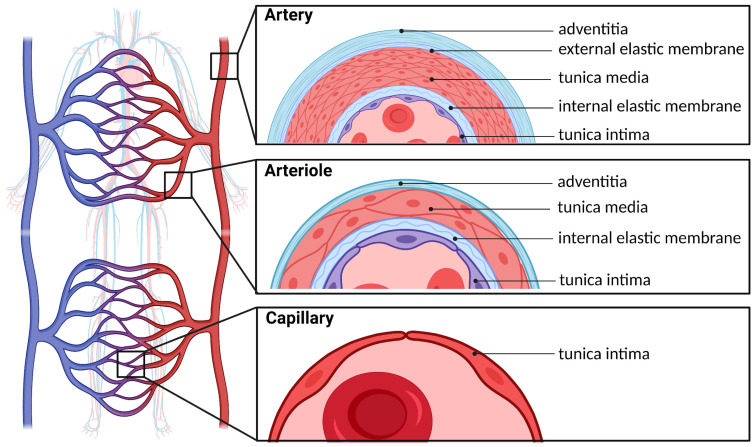
Structure of the arterial system. The circulatory system is a network of arteries and veins connected by capillaries where oxygen and nutrient exchange occurs. The inner lining of arteries, arterioles, and capillaries is known as the tunica intima, which is composed exclusively of endothelial cells. Arterioles and arteries additionally have a series of elastic and muscular layers.

The adult vasculature spans a surface area of approximately 1000 m^2^ and encompasses an arterial and a venous system connected by capillaries (Figure 1) [6]. Importantly, all vessels share a similar basic structure composed of four distinct layers. The innermost layer is known as the tunica intima, consisting of a single layer of endothelial cells that forms the interface with blood. Ensheathing the intima is a smooth muscle layer known as the tunica media, which is innervated by the autonomic nervous system and a major regulator of blood vessel diameter. Large elastic arteries are characterized by a prominent tunica media, whereas capillaries are solely composed of an endothelial monolayer to maximize permeation of oxygen and nutrients into the interstitial space. An adventitial connective tissue forms the tunica externa and communicates with visceral and muscular structures as blood vessels course through various anatomical regions [6].

## 3. Mechanisms of Vascularization

### 3.1. Vasculogenesis

**Figure 2:**
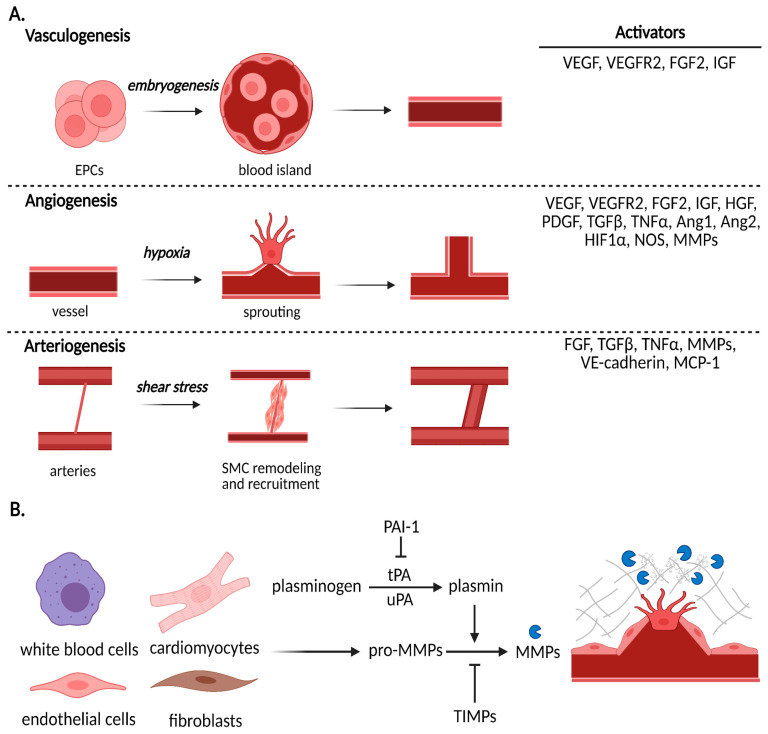
Mechanisms of vascularization and extracellular matrix remodeling. (**A**) Vasculogenesis describes the synthesis of de novo vessels and vasculature that occurs during embryonic development and begins with the differentiation and organization of endothelial progenitor cells. Sprouting angiogenesis is stimulated under hypoxic conditions and is characterized by phalanx, stalk, tip cell migration, proliferation, and tube formation. Arteriogenesis is the process by which shear stress signals for smooth muscle cell recruitment to support an existing vessel between arteries; this vessel then muscularizes to become an established artery. (**B**) An essential process in angiogenesis is extracellular matrix (ECM) remodeling to release growth factor stores from ECM components and promote migration of endothelial cells. A number of cell types contribute to this process in the heart by production of MMPs; their activation is tightly regulated by the plasminogen system and their inhibitors known as tissue inhibitors of metalloproteinases (TIMPs). VEGF, vascular endothelial growth factor; VEGFR2, VEGF receptor 2; FGF2, fibroblast growth factor 2; IGF, insulin growth factor; HGF, hepatocyte growth factor; PDGF, platelet-derived growth factor; TGFβ, transforming growth factor β; TNFα, tumor necrosis factor α; Ang1, angiopoietin 1; Ang2, angiopoietin 2; HIF1α, hypoxia inducible factor 1α; NOS, nitric oxide synthase; MMPs, matrix metalloproteinases; VE-cadherin; vascular endothelial cadherin; MCP1, monocyte chemoattractant protein 1.

By the third week after fertilization, blood vessels begin to form in the yolk sac and then in the growing embryo [7]. Vasculogenesis refers to the initial embryological development of nascent vascular structures, known as blood islands from endothelial progenitor cells (EPCs) (Figure 2A). Morphogenic cues, such as VEGF, trigger differentiation of these precursors and promote formation of blood islands, which then merge into primitive capillary plexuses [8]. Their subsequent growth and expansion to penetrate organs and form an interconnected network is associated with angiogenesis, which we describe next. It is important to first note that while vasculogenesis is most prominently associated with early embryonic development, recent evidence suggests that it is involved in the recruitment of circulating CD34/VEGFR2-positive and bone-marrow-derived angioblasts for in situ growth of blood vessels in post-natal life as well [9]. However, the precise mechanisms by which these angioblasts are stimulated in pathological states and the degree of contribution of this process to the formation of sustainable vasculature remain obscure.

### 3.2. Angiogenesis

Angiogenesis is generally considered the mainstay of neovascularization, where new vessels form from existing vessels, and occurs in many physiological processes such as wound healing, ovulation, and pregnancy. In these contexts, angiogenesis is tightly regulated by an intricate balance between pro- and anti-angiogenic factors. Disturbance of this balance results in uncontrolled blood vessel growth, which is a hallmark pathological feature seen in malignancy, retinopathy, and other disease states.

Activation of the vascular endothelium to switch from a quiescent state to a proliferative state is initiated by an increased local production of nitric oxide (NO) levels, which increases vascular permeability and upregulates expression of VEGF [10]. A series of alterations to intercellular adhesion molecules and cell membrane structure facilitate the extravasation of plasma proteins into the interstitial space. Next, proteases degrade the basement membrane and extracellular matrix (ECM), thereby clearing the path for migrating endothelial cells and, importantly, liberating cryptic adhesion sites and sequestered growth factors. Degradation of the ECM is a complex, highly regulated process. Over twenty identifiable matrix metalloproteinases (MMPs) take part in this step, which are most commonly activated by plasmin and inhibited by tissue inhibitors of metalloproteinases (TIMPs) (Figure 2B) [11,12]. This balance between proteases and their inhibitors is critical, as excessive proteolytic activity is a characteristic of pathological vessel formation in cancer and inflammatory disease.

Following migration and proliferation, tube formation or lumenogenesis occurs. At the cellular level, this process has been shown to occur via budding or cell hollowing. Molecularly, lumen formation is initiated by integrins, while lumen diameter is regulated by contractile status [13]. To this point, the endothelium acquires highly specialized characteristics according to the local tissue needs. For instance, endothelial junctions in capillaries forming the blood–brain barrier are narrow, allowing for controlled permeation of fluid as opposed to those of the glomerular capillaries, which are redundant to allow for filtration. The factors that determine such differentiation of the endothelium are largely unknown; however, the host tissue environment and VEGF signaling likely play a major role. The nascent vessels then undergo three-dimensional organization to form mature vascular networks, which is primarily directed by VEGF, and otherwise known as remodeling or branching [13]. This step occurs either by sprouting towards an angiogenic stimulus, splitting into individual daughter vessels by the formation of trans-endothelial bridges, or intussusceptive insertion of interstitial tissue columns into the lumen of pre-existing vessels. Meanwhile, the structural configuration of the newly shaped vessels composed of an endothelial mono- or double-layer is consolidated by the formation of an ECM and recruitment of peri-endothelial cells. In addition to providing structural support, vascular smooth muscle cells (VSMCs) also inhibit endothelial cell migration and proliferation, thus preventing regression of the nascent vessels.

Interestingly, differentiation of evolving vessels, i.e., artery, vein, or capillary, seems to be influenced by an interplay between external hemodynamic forces and intrinsic molecular signals. Areas of reduced blood flow favor the persistence of capillaries and may even result in complete regression of the primitive vessel if limitation of flow is significant. On the other hand, increased perfusion, pressure, and shear stress induce local recruitment of VSMCs, which lead to arterialization. Basic helix-loop-helix (bHLH) transcription factors appear to play a key role in directing angioblasts to pre-arterial or pre-venous specifications [14]. Additionally, Notch signaling and members of the large ephrin family, along with other tyrosine kinases appear to modulate differentiation of vesicular structures. Longevity of the newly formed vessels is maintained by an ongoing interaction of VEGF with the VEGFR2, P13 kinase, and β-catenin, which induces anti-apoptotic genes and promotes survival [13]. Surprisingly, hemodynamic shear forces favor inhibition of endothelial turnover and, thus, prevent tumor necrosis factor α (TNFα)-mediated apoptosis and vessel regression.

### 3.3. Arteriogenesis

Arteriogenesis is an adaptive phenomenon, which occurs with stenotic vascular lesions and features primary remodeling of existing collateral arteries rather than growth of new vascular structures. As opposed to angiogenesis, which is hypoxia-driven and initiated by the endothelium, arteriogenesis is predominantly stimulated by shear forces. Thus, turbulent blood flow is sensed by the endothelium, which induces the transcription of several genes including FGF2, PDGF-B, and TGFβ [15]. As a result, chloride channels open and adhesion molecules on the endothelium are upregulated, including monocyte chemoattractant protein 1 (MCP1) [16]. Circulating monocytes adhere to and invade arteriolar collaterals, initiating a myriad of inflammatory signals that recruit fibroblasts, platelets, and basophils. The local inflammatory response induces apoptosis in neighboring cells, which facilitates expansion of the collateral vessel diameter up to twenty times [17]. Importantly, the local production of growth factors such as FGF2 induces mitosis of the endothelial and smooth muscle cell layers, again forming a preliminary vascular structure that is remodeled into a final configuration [18]. To underscore that arteriogenesis occurs independent of hypoxia, it has been shown that the distance between ischemic regions and site of collateral vessel formation can be up to 70 cm. [6]. Unlike angiogenesis, arteriogenesis invariably results in competent vasculature capable of sustaining tissue viability; therefore, therapeutic targeting of arteriogenesis may prove to be a rewarding endeavor.

## 4. Preclinical Studies and Clinical Trials

### 4.1. Protein Therapy

Expanding insight into the molecular basis of neovascular formation underscored the critical role of growth factors and provided impetus for early studies, which focused on delivery of recombinant angiopeptides to target ischemic tissue. Table 1 summarizes the outcomes of clinical trials with protein therapy, in addition to gene- and stem-cell-based therapies.

Both VEGF and FGF have a central role in multiple steps of vessel development and differentiation and, therefore, are the most widely studied proteins in the search for novel angiogenic therapies. VEGF isoforms bind to and phosphorylate VEGFR1 and VEGFR2, whereas FGF binds selectively to four major receptors (FGFR1b/c, FGFR2b/c, FGFR3b/c, and FGFR4) on the vascular endothelium [19,20]. The binding of VEGF and FGF to their cognate tyrosine kinase receptor induces diverse downstream signaling pathways including MAPK, P13K, and PLC-γ. Data from preclinical studies showed increased blood vessel growth and collateral-dependent perfusion in animal models of chronic myocardial ischemia with VEGF and FGF therapy [5]. Among other growth factors that have been investigated in animal models of chronic myocardial ischemia are platelet-derived growth factor (PDGF), which positively regulates maturation of vasculature [21]. Additionally, angiopoietin-1 interaction with the Tie-2 receptor has been shown to promote stability of newly formed vessels in preclinical studies [22]. Hepatocyte growth factor (HGF) has well-documented pro-angiogenic roles in post-ischemic and post-infarcted heart failure models as well [23,24]. Although morphogens such as sonic hedgehog, Notch, and Wnt are involved in an array of pathways, taking advantage of their role in blood vessel growth has attracted attention.

A plethora of data from the early clinical trials has largely demonstrated the safety and practicality of administering these growth factors to patients with refractory coronary artery disease such as the phase II VIVA trial and FIRST trial using recombinant VEGF and FGF2, respectively [25,26]. However, it was eventually recognized that the administration of these angioproteins in a clinical setting has resulted in short-lived improvements in collateral dependent perfusion that do not contribute to sustainable clinical benefit. Thus, significant limitations of these recombinant angioproteins were identified; the peptides had a short half-life and required administration of relatively large doses in order to elicit an effect, carrying the risk of significant adverse effects.

### 4.2. Gene Therapy

The delivery of a recombinant gene overtook protein therapy approaches, allowing for persistent expression of the encoded target protein in tissue. Indeed, the myocardium was found to be a suitable substrate for gene transfer, expressing target proteins encoded by viral vectors and non-viral vectors such as naked plasmids and liposome vehicles. Viral vectors tend to employ adenoviridae or retroviridae and are characterized by a high transfection efficiency although potentially immunogenic. The REVASC study was a large phase II randomized clinical trial that reported improved angina symptoms and exercise tolerance following intramyocardial injection of adenoviral-encoded VEGF when compared to optimal medical therapy [30]. However, the possibility of a placebo effect due to lack of blinding and occurrence of complications associated with thoracotomy procedure in four patients were limitations of this study.

Recombinant DNA delivered by means of non-viral vectors are generally more liable to destruction by circulating nucleases, which may shorten the half-life of these genes in target tissue. Nonetheless, many studies have reported meaningful therapeutic benefits with non-viral gene transfer and the lack of an immune reaction permits repeated administration. The Kuopio angiogenesis trial showed that intramyocardial injection of the recombinant VEGF gene on an adenoviral vector during percutaneous coronary angioplasty significantly increased myocardial perfusion when compared to delivery of the VEGF gene using a naked plasmid vector [31]. Moreover, phase I studies evaluating intramyocardial injection of plasmid-encoded VEGF DNA via thoracotomy in patients with end-stage coronary artery disease were associated with improvement of symptoms and blood flow to ischemic territories [27,28,29].

The transfer of human FGF4 bound to an adenovirus (Ad5-FGF4) vector by intracoronary infusion resulted in increased FGF mRNA production at twelve weeks, enhanced collateral dependent perfusion, and lessened the severity of symptomatic angina in patients in the AGENT trial [32]. These promising findings led to initiation of the AGENT-2, -3, and-4 trials, which were designed to assess the ultimate efficacy of Ad5-FGF4 in inducing ischemic myocardial neovascular formation in patients with stable exertional angina controlled with medical therapy (capable of exercising on a treadmill for at least three minutes) and anatomy suitable for, but not in need of, immediate revascularization. Agent-3 and Agent-4 trials, respectively, enrolled 450 and 532 patients and randomly assigned them to receive either placebo or Ad5-FGF4 as an intracoronary injection, the primary endpoint being change in exercise tolerance twelve weeks post-treatment [40]. However, it was eventually found that the treatment offered no significant benefit over placebo, which led to discontinuation of these studies.

Adenoviral transfer and recombinant DNA have, therefore, faced challenges in the clinical setting likely due to breakdown in the circulation, but also includes limitations of cell turnover and risk of immune responses. CRISPR/Cas9 may hold the key to effective gene therapy approaches by offering increased precision and efficiency in comparison to conventional gene targeting approaches. In fact, Huang and colleagues designed a CRISPR/Cas9 system to deplete VEGFR2 in vascular endothelial cells, which was found to block angiogenesis in a mouse model of retinopathy [41]. While these results are recent, they provide the groundwork for future genome editing studies in the field to insert proangiogenic genes or delete inhibitory genes.

### 4.3. Stem Cell Therapy

Certain populations of stem and progenitor cells have the capacity to proliferate and differentiate into vascular components. Upon stimulation, angioblast-derived EPCs mobilize from the bone marrow and localize to sites of endothelial injury, differentiating into mature endothelial cells. Mesenchymal stem cells (MSCs) are another major source of adult stem cells that have been extensively studied for potential applications in cardiac regeneration, due to their reduced risk of immunogenicity and tumorigenicity. Indeed, data from animal studies showed that both bone-marrow-derived EPCs or those isolated from peripheral blood promoted vessel growth in ischemic tissue [42,43]. Subsequently, many clinical trials evaluated various methods of EPC or MSC administration to ischemic tissue.

Numerous early-phase trials of myocardial ischemia provided proof of concept that EPCs or MSCs improved vascularity in ischemic myocardial territories and overall cardiac function [34,35,36,38,39]. They also substantiated that intramyocardial transplantation of autologous bone-marrow-derived cells was a safe, feasible intervention that enhanced myocardial contractility and perfusion while reducing infarct size and cause-related mortality in patients with ischemic heart disease (IHD). Cytotherapy quickly gained popularity as a novel treatment for IHD and was adopted by many centers, thus generating much positive data showing improvement in various functional and symptomatic parameters following innovative methods of administration of MSCs such as NOGA guided delivery. Again, longevity of these therapeutic effects and reproducibility in larger, diverse patient populations was challenging [37,38,39,40,41,42,43,44]. Further investigation revealed that a considerable portion of the injected cells do not survive beyond the first three days and that restoration and replacement of the dying cells was lacking. Moreover, the remaining cells did not appear to conform into functional tissue that integrated into the injured myocardium. Contrarily, a trophic effect was often observable in the tissue at sites of cell transplantation.

Another caveat that impedes this cell-based therapy approach is the lack of uniform measures for characterization of cells and determination of their secretory properties. The general consensus is the use of flow cytometry to identify cells by specific markers. Such classifications may only be useful for the theoretical study of the functional behavior of cells with similar structural characteristics. In reality, however, stem and progenitor cells exhibit tremendous plasticity and may rapidly alternate phenotypes in different environments [45]. Exposure of pre-transplanted stem cells to hypoxic conditions stimulates ischemic tissue and induces the expression of many angiogenic factors and survival proteins that improve therapeutic efficacy. Growth factors such as VEGF, placental growth factor, and stem cell factor can mobilize endogenous bone-marrow-derived cells and direct their differentiation into specialized cell types with the capacity to express angiogenic factors. Another exciting approach involves engineering tissue using pluripotent stem cells and vascular progenitor cells induced to differentiate into contractile and vascular elements, respectively. Among other creative strategies that have been employed to promote survival of autologous stem cells are localized ultrasound-targeted microbubble destruction of tissue at the transplantation site to create a void that facilitates growth of the nascent cells and the use of grafts composed of cells and an ECM [46].

### 4.4. Extracellular Vesicle Therapy

The field of stem-cell-derived extracellular vesicles (EVs) advanced following findings that the therapeutic effects of cell-based therapies were mediated by paracrine actions [47,48]. The cardioprotective effects of EVs have since been well characterized in small and large animal models [49,50]. While EVs carry a diverse cargo of proteins, RNAs, and lipids that may mediate many pathways related to cardiac remodeling, their pro-angiogenic effects have been corroborated in vivo and in vitro. Proteomic characterization further supports the enrichment of pro-angiogenic pathways in EVs, which was shown to be regulated by NFκB signaling in an elegant study by Anderson and collaborators [51,52].

Although the field of stem-cell-derived EVs has gained much popularity amongst the research community, the translation of this therapeutic modality to the clinic has only just begun in the setting of cardiovascular diseases. Phase II and phase I studies with MSC-EV treatment for acute ischemic stroke and multiple organ dysfunction syndrome (MODS), respectively, are currently in progress (NCT03384433, NCT04356300). Challenges in successful translation reside in the lack of standardized characterization methods and targeted delivery to injured tissue. However, bioengineering strategies will be invaluable in customizing EV content in addition to improving delivery and biodistribution to maximize the potential of stem-cell-derived EV therapies.

## 5. Future of Therapeutic Angiogenesis

### 5.1. Patient Selection

Clinical trial eligibility criteria are essential in mitigating potential variables such as age, gender, disease state, and so forth to ensure safety, mitigate confounding factors, and isolate the effects of the treatment. Early clinical trials aiming at revascularization often targeted patients with significant severity of disease, who had previously undergone multiple failed interventions. This limited patient cohort suggests an inherent deficiency in the mechanisms needed for blood vessel growth, conferring a lack of responsiveness to growth factors and other therapies [53,54]. Additionally, many medications widely used in practice have well-documented anti-angiogenic properties including atorvastatin, spironolactone, captopril, and aspirin [55,56,57,58,59,60]. Experimental animal models may, therefore, be necessary to evaluate pro-angiogenic treatments at various time points of the disease, in addition to studying preventative measures prior to induction of the disease model. Such studies will shed light on the effectiveness of the treatment in relation to the time it was administered and the severity of the disease. Bioinformatics analysis to examine the database of clinical trial data may also be useful in evaluating the relationships between disease state, dose and delivery, and outcomes.

Additionally, lack of standardized endpoints to assess outcomes in patients enrolled in therapeutic angiogenesis trials makes interpretation difficult. Indeed, the gold standard remains the demonstration of new vessels or improved blood flow using imaging techniques or perfusion studies, respectively. However, the longevity of this vasculature and clinical significance with respect to improving quality of life and survival has been subject to controversy. Sun et al. employed simulations and statistical analysis to evaluate multiple endpoints in phase II acute heart failure clinical trials. They found that the average Z score, which considers the average among all endpoints, is most powerful [61]. Of course, the authors importantly note that sample size may require the application of different statistical methods and criteria.

### 5.2. Comorbidities

Preclinical studies evaluating the efficacy of therapeutics in the setting of CVDs often show promise but have little to no success when translated to the clinical setting. This discrepancy may be in part attributed to underlying comorbidities in humans that are unaccounted for in laboratory animal models. This issue is further highlighted by the high prevalence of obesity and diabetes in the U.S. population, which is, respectively, 40 and 10% as reported by the Centers for Disease Control and Prevention [62,63].

**Figure 3:**
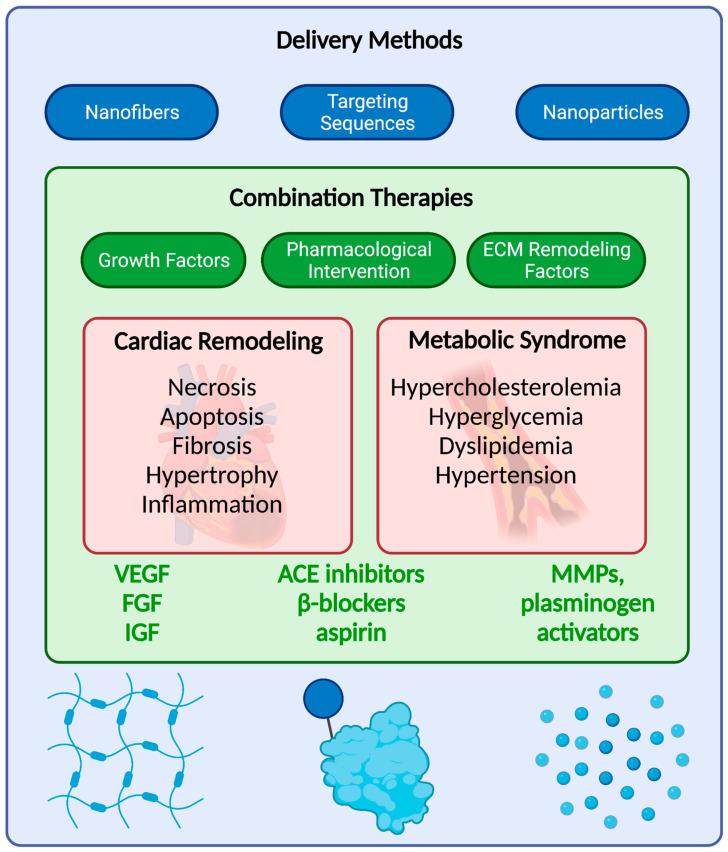
Future challenges and prospects in therapeutic angiogenesis. Cardiac remodeling is characterized by widespread maladaptive changes that adversely affect the structure and function of the heart; these events are further exacerbated by underlying comorbidities such as metabolic syndrome. Combination therapies have the potential to mediate the widespread changes and enhancing revascularization. Furthermore, bioengineering methods may play a valuable role in controlling the release of signaling factors, improving myocardial targeting, and encapsulating many factors.

Indeed, four weeks of a high-fat diet or glucose intolerance has been associated with markedly increased expression of anti-angiogenic factors endostatin and angiostatin, increased oxidative stress and additional signaling abnormalities, which likely have a major effect in diminishing the angiogenic response to growth factors or cell therapy, or the angiogenic process in general in both animal models and in patients [64,65,66]. A recent study by our lab indeed found disparate gene expression and paradoxical angiogenic signaling between a chronic ischemia swine model with and without metabolic stress, when treated with EVs [67]. Therefore, future preclinical work must compare functional, cellular, and molecular effects of therapeutic treatments targeting angiogenesis in disease states with additional risk factors [68]. The widespread maladaptations that occur during cardiac remodeling, combined with underlying risk factors, also highlight the growing need for a more comprehensive or versatile treatment approach, such as combination therapies (Figure 3). In fact, therapeutic interventions such as glucose control seem to improve the potential of angiogenesis and collateral vessel growth in animal models [69].

### 5.3. Combination Therapies

The combination of proteins, genes, and/or cells is sound rationale to overcome the shortcomings of monotherapies. Indeed, co-administration of VEGF and PDGF, FGF2 and PDGF, or VEGF and FGF2 were found to improve revascularization compared to controls in ischemic tissues in vivo [70]. Bai and collaborators investigated single, binary, and ternary combination of growth factors with VEGF, FGF2, and bone morphogenic proteins 2 (BMP2). Together, these factors significantly improved endothelial cell angiogenesis in vitro and chorioallantoic membrane angiogenesis in vivo, with reduced concentrations of each factor [71]. Methods of modifying gene expression in BM-MSCS have had similar success; BM-MSCs overexpressing HGF or ANG1 were shown to increase vascularity in the ischemic myocardial territory [72]. The transfer of endothelial nitric oxide synthase (eNOS) to BM-MSCs using minicircle plasmid DNA also enhanced angiogenic capacity of these cells [73].

Preconditioning or priming cells with a given stimuli has also emerged to manipulate cellular cargo in place of single-gene transfections. This approach has the ability to tune the cell’s contents at a much larger scale. The review by de Cássia Noronha et al. describes potential stimuli such as hypoxia, cytokine exposure, and nutrient or drug administration applied while culturing cells, and their therapeutic effects in animal models [45]. As the field of stem-cell-based therapies has been impeded by clinical findings with short-lasting improvements that are not sustained in the long-term, the field of stem-cell-derived EVs has conversely risen. Certainly, preconditioning or transfecting cells and isolating their EVs has potential in future clinical trials. A recent study by Sun and collaborators found that exosomes derived from hypoxia inducible factor 1 α (HIF1α)-overexpressing MSCs resulted in cardioprotection of a rat myocardial infarction model by inducing angiogenesis [74]. Omics studies will play a major role in characterizing EV cargo and ensure standardization for potential large-scale application. Certainly, laboratory animal models may not perfectly replicate the conditions inevitably associated with the clinical setting such as interfering comorbidities, medications, and refractory disease; however, creative and comprehensive preclinical study designs that considers comorbidities, combination therapies, and delivery systems are increasingly imperative.

### 5.4. Delivery

Ongoing studies aim to provide an optimal mode of delivery that does not necessitate repeated invasive procedures and ensure sustained tissue expression of the therapeutic substance. Indeed, thoracotomies and other surgical methods of delivery carry significant risks and prohibit effective control groups for clinical trials. These limitations prompted interest in employing cutting edge imaging modalities in the development of less invasive methods of administration of the gene-vectors to ischemic myocardial territories. Data from clinical trials showed that NOGA guided delivery of plasma encoded VEGF in patients with chronic symptomatic angina who are not candidates for conventional means of revascularization effectively improved Canadian Cardiovascular Society (CCS) angina class, while being well tolerated [75].

Novel delivery methods via nanofibers, nanoparticles, and targeting sequences may also be critical in overcoming these challenges. Among the nanofiber materials available, hydrogels are particularly intriguing due to their water content that is compatible with bodily tissues and support slow diffusion of bioengineered contents. An alginate-based gel containing VEGF found a stable release of the growth factor over one month in vitro and improved angiogenesis in a hindlimb ischemic model in vivo [76,77]. Of course, many polymer options can and should be explored in the development of a delivery system that maximizes and extends the angiogenic signal, such as collagens, gelatins, fibrins, peptides, and matrigels.

Nanoparticles may be sourced synthetically or from bioparticles, such as extracellular vesicles themselves; natural bioparticles may be a safer mode that avoids the risk of immunogenicity. Nonetheless, these particles—whether synthetic or natural in nature—are capable of being carriers of genes, proteins, drugs, and other molecules. The advantage to this method is protecting the molecule from potential degradation until fusion with recipient cells; furthermore, size and membrane content can be adjusted to improve delivery towards target tissue. In fact, in vivo biopanning approaches have identified cardiac-specific targeting peptides. Separate studies identified such targeting peptides, which were referred to as cardiac homing peptide (CHP) or ischemic myocardium-targeting peptide (IMTP), and conjugated them to exosomal membrane proteins [78,79]. They then proceeded to study the actions in vivo into animal models of MI and found improved delivery, biodistribution, and cardioprotection in the myocardium compared to controls [80,81].

A novel delivery platform was recently developed by Wang, designed to optimize delivery of growth factors and known as coacervate [82,83]. The coacervate forms due to electrostatic interactions that essentially polymerize into tiny oil droplets and mimic the structure of heparin along with its capability to bind many factors at once. Therefore, the coacervate has potential to deliver multiple growth factors, which may be of particular value in stimulating angiogenesis. In vitro examination supported the loading ability and controlled release of this platform; meanwhile, an in vivo study with FGF2 significantly improved angiogenesis compared to free FGF2 [82,83]. A recent study by Xiao and collaborators supported previous findings, where FGF2-loaded coacervate significantly enhanced wound healing via cell proliferation, VEGF secretion, and increased CD31 and αSMA density [84].

## 6. Conclusions

Ischemic heart disease is the most prevalent and deadly disease worldwide. While current standards of care have undoubtedly improved outcomes, there is consensus that novel therapies employing inherent mechanisms of revascularization are the next frontier in the management of this disease. Elucidation of angiogenic processes and their underlying mechanisms has provided key insights into activators and targets to stimulate angiogenesis. Protein, gene, and cell-based therapies have been developed; however, their translation from animal models to clinical trials have largely been disappointing. Challenges in patient selection, endpoint measures, and the prevalence of comorbidities have confounded results and interpretation. However, bioinformatic approaches and bioengineering strategies may overcome such challenges by determining optimal statistical methods that account for multiple endpoints, and improving delivery and biodistribution of factors to the damaged tissue. Combination therapies, furthermore, hold promise in mediating multiple pathways and maximizing therapeutic effects.

## Figures and Tables

**Table 1:** Clinical outcomes with angiogenic therapies.

Agent	Study Design (Disease; Delivery; Dose; Number of Patients)	Outcome	Ref.
**Protein Therapy**			
VEGF	CAD; IC day 0 and IV day 3,6,9; 17 ng/kg/min or 50 ng/kg/min; *n* = 178)	No improvement in exercise time 60 days post treatment	[25]
FGF	CAD; IC; single injection of 0, 0.3, 3, or 30 µg/kg; *n* = 337	Exercise tolerance and angina symptoms improved at 90 days; no difference at 180 days	[26]
	CAD; IC via heparin-alginate slow-release device; 1 or 10 µg; *n* = 8	Exercise tolerance and myocardial perfusion showed a trend toward improvement at 90 days, but not at 180 days	[4]
**Gene Therapy**			
VEGF	CAD; IM 10×; 200 µg supplemented with 6 g l-arginine per day for 3 months; *n* = 19	Improved anterior wall perfusion and anterior wall contractility at 3 months	[27]
	CAD, IM; 125 or 250 µg; *n* = 15	Angina was significantly reduced and myocardial perfusion was improved	[28]
	Angina, IM, 200 µL at 10 sites; *n* = 30	Myocardial perfusion reserve significantly increased at 3 months and 12 months compared to baseline, although no significance between 3 and 12 months.	[29]
	IHD, IM, 4 × 10^10^ pfu, *n* = 67	Total exercise duration and time were improved at 12 and 26 weeks	[30]
	IHD, IC, 2 × 10^10^ pfu, *n* = 103	Myocardial perfusion was significantly improved at 6 months; no changes in minimal lumen diameter nor % of diameter stenosis were also reported	[31]
FGF	Angina, IC, 5 different dose groups, *n* = 79	Increased exercise time at 4 weeks	[32]
	CLI; intramuscular; 4 mg at day 1, 15, 30, and 45; *n* = 125	Complete healing of at least one ulcer in the treated limb at week 25; treatment also significantly reduced the risk of all amputations by two-fold	[33]
**Stem-Cell Therapy**			
BM-MSC	MI; IC; day 6 post-MI on average; 7 × 10^5^ cells; *n* = 101	LVEF was increased at 6 months; no change in LV EDV nor infarct size was observed.	[34]
	CAD; transendocardial injection; 1 × 10^8^; *n* = 92	LV ESV nor maximal oxygen consumption were improved at 6 months	[35]
	(MI; IC; 24.6 ± 9.4 × 10^8^ nucleated cells, 9.5 ± 6.3 × 10^6^ CD34+ cells, and 3.6 ± 3.4 × 10^6^ hematopoietic cells ~4.8 days post-MI; *n* = 60)	LVEF was improved at 6 months, but was not significant at 18 months	[36]
CPC	IHD; IM; injections at 17 sites; *n* = 315	No significant improvements in primary endpoints of MLHFQ score, 6 min walk distance; LV ESV and LV EF at 39 weeks	[37]
	IHD; IM; 600 × 106 to 1200 × 106 cells; *n* = 319	LVEF was improved with reduction in LV ESV, and improved 6-min walk distance	[38]
BMC or CPC	MI, IC, mean of 22 × 10^6^ CPC or 205 × 10^6^ BMC, *n* = 75	BMC treatment significantly increased LVEF compared to CPC and control groups at 3 months.	[39]

VEGF, vascular endothelial growth factor; FGF, fibroblast growth factor; BM-MSC, bone-marrow-derived mesenchymal stem cells; CPC, cardiopoietic stem cells; CAD, coronary artery disease; CLI, chronic limb ischemia; IHD, ischemic heart disease; MI, myocardial infarction; IC, intracoronary; IV, intravenous; IM, intramyocardial; LVEF, left ventricular ejection fraction; EDV, end diastolic volume; ESV, end systolic volume; EF, ejection fraction.
